# PEMFC model identification using a squeezenet developed by modified transient search optimization algorithm

**DOI:** 10.1016/j.heliyon.2024.e27555

**Published:** 2024-03-09

**Authors:** Rulin Duan, Defeng Lin, Gholamreza Fathi

**Affiliations:** a School of Computing, Guangdong Vocational Institute of Public Administration, Guangzhou, 510800, Guangdong, China; b School of Psychology, South China Normal University, Guangzhou, 510631, Guangdong, China; c Department of Electrical Engineering, Power & Water University of Technology (PWUT), Tehran, Iran

**Keywords:** Proton exchange membrane fuel cells, Model identification, Squeezenet, Modified transient search optimization algorithm, Sum of squared error, Output voltage

## Abstract

Proton Exchange Membrane Fuel Cells (PEMFCs) are promising sources of clean and renewable energy, but their performance and efficiency depend on an accurate modeling and identification of their system parameters. However, existing methods for PEMFC modeling suffer from drawbacks, such as slow convergence, high computational cost, and low accuracy. To address these challenges, this research work proposes an enhanced approach that combines a modified version of the SqueezeNet model, a deep learning architecture that reduces the number of parameters and computations, and a new optimization algorithm called the Modified Transient Search Optimization (MTSO) Algorithm, which improves the exploration and exploitation abilities of the search process. The proposed approach is applied to model the output voltage of the PEMFC under different operating conditions, and the results are compared with empirical data and two other state-of-the-art methods: Gated Recurrent Unit and Improved Manta Ray Foraging Optimization (GRU/IMRFO) and Grey Neural Network Model integrated with Particle Swarm Optimization (GNNM/PSO). The comparison shows that the proposed approach achieves the lowest Sum of Squared Errors (SSE) and the highest accuracy, demonstrating its superiority and effectiveness in PEMFC modeling. The proposed approach can facilitate the optimal design, control, and monitoring of PEMFC systems in various applications.

## Nomenclature

Symbol: Explanation Unit
$E_{\text{Nernst}}$: Nernst voltage V
$T_{\text{FC}}$: The PEMFC operating temperature K
$P_{H_{2}}$: The partial pressure of the hydrogen atm
$P_{O_{2}}$: The partial pressure of oxygen atm
A: The active area of the membrane cm2
$I_{\text{FC}}$: The current of the fuel cell A
$R_{\text{ha}}$: The relative humidity of the anode electrodes
$R_{\text{hc}}$: The relative humidity of the cathode electrodes
$P_{a}$: The input partial pressures for the positive electrodes atm
$P_{c}$: The input partial pressures for the negative electrodes atm
$\mathrm{Ln}\left(P_{H_{2}O}\right)$: The saturation vapor pressure in a Proton Exchange Membrane Fuel Cell
$T_{F}$: The cathode's operating temperature K
$V_{\text{out}}$: The actual output voltage of the PEMFC V
$E_{\text{cons}}$: Concentration voltage losses V
$E_{\text{act}}$: Activation voltage losses V
$E_{\Omega }$: Ohmic voltage drop V
$\gamma_{i|i}$: The resultant coefficients
$C_{O_{2}}$: The saturation of oxygen at the negative electrode catalytic interface $\text{mol}/cm^{3}$
$C_{H_{2}}$: The saturation of hydrogen at the negative electrode catalytic interface $\text{mol}/cm^{3}$
β: Coefficient
J: The standard densities A/m2
$J_{\max}$: The maximum flow densities A/m2
$R_{m}$: The membrane resistance Ω
S: The surface area of the membrane cm2
l: The thickness of the membrane cm
$\rho_{m}$: The resistivity of the membrane Ω.m
λ: The control parameter
$R_{c}$: The connection resistance Ω
SSE: The Sum of Squared Errors
M: The sampling amount of empirical information
GRU/IMRFO: Gate Recurrent Unit and Improved Manta Ray Foraging Optimization
GNNM/PSO: Grey Neural Network Model integrated with Particle Swarm optimization
PEMFCs: Proton Exchange Membrane Fuel Cells
CNN2D: Two-Dimensional Convolutional Neural Network
LOESS: Locally Weighted Scatterplot Smoothing
MI: Mutual Information
FDB: Fitness-distance balance
CFD: Computational Fluid Dynamic
LHS: Latin hypercube sampling
ANN: Artificial Neural Network
NSGA-II: Non-Dominated Sorting Genetic Algorithm
MOO: Multi-Objective Optimization
SOO: Single-Objective Optimization
DHOA: Deer Hunting Optimization Algorithm
MTSO: Modified Transient Search Optimization
PEM: Proton Exchange Membrane
TDL: Time Delay Line -

## 1 Introduction

### 1.1 Background

Proton Exchange Membrane Fuel Cells (PEMFCs) have emerged as a very promising technology for the conversion of energy in a clean and efficient manner. Electric vehicles provide several benefits, including little pollutants, subdued operational noise level, and notable energy economy. These advantageous attributes assume them well-suited for diverse applications, encompassing transportation, stationary power generating, and portable electronics [1].

In order to maximize the potential of PEMFCs and achieve their best use, it is essential to possess precise models that effectively reflect the complex electrochemical and transport phenomena, taking place inside the fuel cell [2]. These models are used as important instruments for comprehending the intricate behavior of PEMFCs, enhancing their design and operation, and forecasting their performance under various operating scenarios [3].

Nevertheless, the problem of precisely determining the ideal model parameters for PEMFCs poses a significant challenge [4]. The intricate nature of the system, along with uncertainty surrounding material characteristics, operational circumstances, and a multitude of internal and external elements, presents considerable challenges [5]. Inaccurate forecasts and inferior performance may result from an erroneous estimate of the model parameters [6].

In recent years, there has been a significant research effort focused on exploring various optimization techniques with the aim of facilitating the determination of optimum model parameters for PEMFCs [7]. The primary objective of these algorithms is to reduce the discrepancy between seen or measured data and the anticipated outcomes generated by the model, hence enhancing the precision of the model [8].

A range of optimization strategies, including Enhanced Bald Eagle Algorithm [9], Bi-Subgroup Optimization Algorithm [10], Collective Animal Behavior Algorithm [11], have been used to conduct a search for the most optimum parameter values [12]. The algorithms use iterative techniques in order to investigate the parameter space and identify the collection of parameters that exhibit the highest degree of agreement with the experimental data or reduce the disparity between anticipated and observed behavior [13].

The primary aim of these optimization techniques is to improve the precision and dependability of PEMFC models, hence facilitating a more comprehensive comprehension and management of the fuel cell system [14]. Through the process of refining the model parameters, researchers and engineers have the opportunity to acquire valuable knowledge about the intricate processes, taking place inside PEMFCs. This, in turn, may result in the development of more effective design strategies, heightened performance levels, better durability, and decreased expenses [15].

### 1.2 Related works

The use of neural networks in conjunction with metaheuristics, as opposed to only relying on optimization methods, offers superior advantages in the modeling of PEMFC. This approach enables a more precise and efficient determination of the best parameters for the model. The integration of neural networks with metaheuristic algorithms may effectively capitalize on the respective advantages of both methodologies, leading to an enhanced precision and efficacy in the process of model identification. For example, Tan et al. [16] proposed an active model, which was system-level, comprising electric stack, hydrothermal management system, hydrogen supply system, and the gas supply system. It is noteworthy, it was conducted in accordance with Simulink and MATLAB. The simulation data was used to fit the electric stack model through the neural network. Various operating circumstances' impacts on performance of system and stack power of the PEMFC were analyzed [17]. In order to combine performance of the system and reactor's power for expressing combined efficiency index, an algorithm was presented, called Particle Swarm Optimization. In addition, the primary purpose of it was optimization of system's efficacy and power density with several intentions. Eventually, the system's efficiency and the power density increased by 12.8% and 1.33, respectively, decreased the power of parasite, and increased the performance output to the maximum amount.

Tao et al. [18] proposed Gate Recurrent Unit (GRU), Improved Manta Ray Foraging Optimization (IMRFO), and a hybrid deep learning design in accordance with Two-Dimensional Convolutional Neural Network (CNN2D) for efficiency degradation forecasting of the PEMFC. In the beginning, the Locally Estimated Scatterplot Smoothing (LOESS) and Mutual Information (MI) were utilized to process the data beforehand. The purpose was to enhance quality of sample and decrease the impact of noisy and unimportant data for prediction of the model. Next, CNN2D was utilized to thoroughly investigate the non-linear degradation traits within the data. Third, three methods, namely Exponential Weight Coefficient, Fitness-distance balance (FDB), and half uniform initialization, were incorporated into the algorithm to prevent it from getting trapped in local optima. In the end, in order to achieve the concluding outcomes and forecast the degradation data, the enhanced MRFO optimized the model of GRU. The findings illustrated that the MAE and RMSE are, in turn, 0.0042 and 0.072; moreover, the forecasting accuracy of the suggested model is 99.79%. Eventually, the approach can enhance robustness, accuracy, and reliability of efficacious degradation forecasting of Proton Exchange Membrane Fuel Cell and efficiently discover the deep attributes of the data.

Chen et al. [19] proposed a method called Grey Neural Network Model (GNNM) integrated with Particle Swarm optimization (PSO); additionally, in order to predict the PEMFC degradation underwent various operating condition, the Moving Window approach was suggested. The suggested approach took into account the impact of inlet temperature, load current, inlet relative humidity, and inlet hydrogen pressure. Grey Neural Network constituted the degradation forecasting system of PEMFC. Particle Swarm Optimization (PSO) optimized threshold and initial weight of GNNM. The PSO-GNNM, which was optimized, was trained iteratively in accordance with the Moving Window approach by many novel evaluated data. The effect of various sizes of Moving Window on the degradation forecasting of the Proton Exchange Membrane Fuel Cell was examined under the load current. After that, system of the Adaptive Neuro-Fuzzy inference approach and the suggested approach was compared, and the comparison between them was discussed. After performing three aging experiments on PEMFCs under various circumstances, it was found that the suggested approach is capable of precisely predicting the deterioration of PEMFCs in a range of applications.

Yu et al. [20] proposed a method to enhance efficiency of PEMFC that integrates intelligent optimization algorithms, Artificial Neural Networks (ANN), and Computational Fluid Dynamic (CFD). In the beginning, a 3-D fine-mesh field of flow and a PEMFC CFD that enjoyed a three-dimensional (3-D) multiphase was made. The optimality decision elements were derived from the primary structural features of the fine-mesh flow field. The Latin Hypercube Sampling (LHS) experimental method was utilized to select the sampling points. The CFD model calculated oxygen uniformity index of sample arguments and density of power. It was utilized to exercise the replacement model of the Artificial Neural Network (ANN). By the use of Non-Dominated Sorting Genetic Algorithm (NSGA-II) and Genetic Algorithm (GA), Multi-Objective Optimization (MOO) and Single-Objective Optimization (SOO) were, in turn, conducted. According to the findings, utilizing the surrogate models of ANN led to a high accuracy of forecasting. The highest density of power in SOO rose by 7.564% that was 0.562% greater compared to MOO case. On the other hand, the total pressure of drop in cathode of SOO was larger compared to the base case and MOO. Moreover, the water removal capacity, the concentration of oxygen, and the uniformity index of oxygen were finer compared to SOO. In order to enhance the entire efficiency of the PEMFC, the enhanced flow field construction, which was optimized by MOO, was known to be more profitable.

Yuan et al. [21] proposed an optimal technique to identify elements of a Proton Exchange Membrane Fuel Cell (PEMFC) and enhance the accuracy of the model. An enhanced model in accordance with Deer Hunting Optimization Algorithm (DHOA) was conducted to the Convolutional Neural Network for the aim of detecting PEMFC elements. In fact, this approach was carried out to extend the efficiency of the approach to estimate elements of the PEMFC. Then, the approach was verified according to four conditions of operation. The outcomes acknowledged that using the suggested approach provided the scholars with better accuracy.

### 1.3 Motivation and novelty

Each existing method for PEMFC model identification had limitations in terms of accuracy and efficiency. Therefore, there is still a need for an improved approach that can overcome these limitations and provide more reliable models. This research work proposes an improved approach for model identification of PEMFCs using a modified Squeezenet model and the Modified Transient Search Optimization Algorithm.

The motivation behind this research is to address the research gap by proposing an improved approach for model identification of PEMFCs using a modified metaheuristic. The primary focus of the paper is to address a significant issue within the renewable energy sector, specifically pertaining to the modeling and optimization of the performance and efficiency of PEMFCs. PEMFCs are electrochemical devices that convert chemical energy into electrical energy.

In order to tackle this problem, the paper introduces an enhanced approach that combines a modified version of the SqueezeNet model, a deep learning architecture renowned for its ability to reduce parameters and computations, with a novel optimization algorithm known as the Modified Transient Search Optimization (MTSO) Algorithm. This combined approach significantly enhances the exploration and exploitation capabilities of the search process. By utilizing empirical data and conducting comparisons with two other cutting-edge methods (Gated Recurrent Unit and Improved Manta Ray Foraging Optimization, and Grey Neural Network Model integrated with Particle Swarm Optimization), the paper effectively demonstrates the superiority and effectiveness of the proposed approach in the modeling of PEMFCs.

This article discusses the difficulties associated with modeling PEMFC, such as the complexity of the system, nonlinearity, uncertainty, and the lack of reliable data. It presents an innovative approach that combines deep learning and meta-heuristic optimization to achieve precise and robust PEMFC modeling. The effectiveness of this approach is evaluated by the use of various datasets and performance metrics, demonstrating its superiority over existing techniques in terms of convergence speed, computational cost, and accuracy.

The article offers valuable insights for future research and development in PEMFC modeling and optimization, suggesting potential extensions and improvements. Current methods for PEMFC modeling suffer from drawbacks, such as slow convergence, high computational cost, and low accuracy. Therefore, a new method is required that strikes a balance between simplicity and accuracy, gets adapted to changing operating conditions, and harnesses the potential of deep learning and meta-heuristic optimization, which are emerging tools for solving complex and nonlinear problems. In conclusion, this paper presents significant contributions and innovations.

- Firstly, the paper introduces an improved methodology for modeling PEMFC by integrating a modified version of the SqueezeNet model and a novel optimization algorithm known as the MTSO Algorithm.
- Secondly, the paper showcases the superiority and efficacy of the proposed approach in PEMFC modeling through a comprehensive comparison with empirical data and two other cutting-edge methods.
- Lastly, this paper makes a valuable contribution to the progress of knowledge and technology in the realm of PEMFC modeling and optimization. It also offers insightful implications and directions for future research and development in this field.

## 2 System modelling

The PEMFCs require a thorough understanding and analysis of the various physical and electrochemical processes that take place within the cell. These processes are interconnected, and they can collectively determine the overall performance of the fuel cell. The behavior and dynamics of PEMFCs encompass a range of phenomena, including fluid flow, heat transfer, electrochemical reactions, mass and proton transport, and thermodynamics. Each of these processes plays a vital role in the functioning of the fuel cell and needs to be accurately represented in the model.

According to the aforementioned explanations, it can be said that a PEMFC is a kind of fuel cell used for the direct conversion of chemical energy into electricity, exhibiting a notable level of efficiency. Due to the absence of an internal reformer, the primary fuel used in this device is pure hydrogen. Hence, the primary chemical process, occurring in a PEMFC, can be mathematically defined as follows:

(1) $$O_{2}+{2H}_{2}\to 2H_{2}O+Heat+Electricity$$

The reactions, occurring at the anode and cathode sides, are as follows, respectively:

(2) $$H_{2}\to 2H^{+}+2e^{-}$$

(3) $$O_{2}+4H^{+}+4e^{-}\to 2H_{2}O+Heat$$

In a Proton Exchange Membrane Fuel Cell (PEMFC), the open circuit voltage, commonly referred to as the Nernst voltage, is obtained by the use of the equation that follows:

(4) $$E_{Nernst}=1.229-8.5\times 10^{-4}\left(T_{FC}-298.15\right)+4.31\times 10^{-5}{\times T}_{FC}\times \left[\ln\left(P_{H_{2}}\right)+0.5\times \ln\left(P_{O_{2}}\right)\right]$$

where, $T_{FC}$ describes the PEMFC's operating temperature, and $P_{H_{2}}$ is the partial pressure of the hydrogen, and $P_{O_{2}}$ is the partial pressure of oxygen that are mathematically obtained as follows:

(5) $$P_{H_{2}}=\frac{R_{ha}\times P_{H_{2}O}}{2}\left[\frac{1}{\frac{R_{ha}\times P_{H_{2}O}}{P_{a}}\times e^{\frac{1.635I_{FC}/A}{T^{1.334}I_{FC}}}}-1\right]$$

(6) $$P_{O_{2}}=R_{hc}\times P_{H_{2}O}\left[\frac{1}{\frac{R_{hc}\times P_{H_{2}O}}{P_{c}}\times e^{\frac{1.635I_{FC}/A}{T^{1.334}I_{FC}}}}-1\right]$$

In this context, the symbol A denotes the active area of the membrane. $I_{FC}$ represents the current of the fuel cell. $R_{ha}$ and $R_{hc}$ describe the relative humidity of the anode and cathode electrodes, respectively. $P_{a}$ and $P_{c}$ indicate the input partial pressures for the positive and negative electrodes, respectively.

The mathematical modeling and determination of the saturation vapor pressure in a Proton Exchange Membrane Fuel Cell (PEMFC) is achieved using the following procedure:

(7) $$\mathrm{Ln}\left(P_{H_{2}O}\right)=-2.18+0.0295\times \left(T_{FC}-273.15\right)-9.18{\times 10}^{-5}T_{c}^{2}+1.4{\times 10}^{-7}T_{c}^{3}$$

The variable $T_{F}$ is used to denote the cathode's operating temperature (in degrees Celsius). The voltage achieved for the PEMFC tends to be lower than the Nernst voltage. The actual output voltage of the PEMFC is determined by multiple voltage losses, including concentration, activation, and Ohmic voltage drop (($E_{cons})$, ($E_{act})$, and ($E_{\Omega })$).

(8) $$V_{out}=E_{N}-E_{\Omega }-E_{cons}-E_{act}$$

The losses of voltage described above have been defined in the following manner.

### 2.1 The activation voltage drop

(9) $$E_{act}=-\left[\gamma_{1}+\gamma_{2}{\times T}_{FC}+\gamma_{3}\times T_{FC}\times \ln\left(C_{O_{2}}\right)+\gamma_{4}\times T_{FC}\times \ln\left(I_{FC}\right)\right]$$

where, $\gamma_{i|i=1,3,4}$ represents the resultant coefficients, and:

(10) $$\gamma_{2}=4.3\times 10^{-5}\;\ln\left(C_{H_{2}}\right)+2.1\times 10^{-4}\;\ln\left(A\right)+2.9\times 10^{-3}$$

The saturation of oxygen ($C_{O_{2}}$) and hydrogen ($C_{H_{2}}$) at the interface of catalytic negative electrode (expressed in $mol/cm^{3}$) is attained by the following process:

(11) $$C_{O_{2}}=\frac{P_{O_{2}}}{5.1\times 10^{6}}\times e^{\frac{498}{T_{FC}}}$$

(12) $$C_{H_{2}}=\frac{P_{H_{2}}}{1.1\times 10^{6}}\times e^{\frac{-77}{T_{FC}}}$$

### 2.2 The concentration voltage drop [22]

(13) $$E_{cons}=-\beta \times \ln\left(J_{\max}-\frac{J}{J_{\max}}\right)$$

Such that, β, J, and $J_{\max}$ represent a coefficient, the standard, and the maximum flow densities.

### 2.3 The Ohmic voltage drop

(14) $$E_{\Omega }=\left(R_{m}+R_{c}\right)\times I_{FC}$$

Such that,

(15) $$R_{m}=\frac{\rho_{m}\times l}{S}$$

Such that, S represents the surface area of the membrane in squared centimeters ($cm^{2}$), the variable l denotes the thickness of the membrane, and $\rho_{m}$ is the resistivity of the membrane. The value of $\rho_{m}$ may be determined using the following equation:

(16) $$\rho_{m}=\frac{181.6\left[1+0.062{\left(\frac{T_{FC}}{303}\right)}^{2}{\times \left(\frac{I_{FC}}{S}\right)}^{2.5}+0.03\left(\frac{I_{FC}}{S}\right)\right]}{\left[\lambda -0.063-3\left(\frac{I_{FC}}{S}\right)\right]\times e^{\frac{T_{FC}-303}{T_{FC}}}}$$

**Fig. 1:**
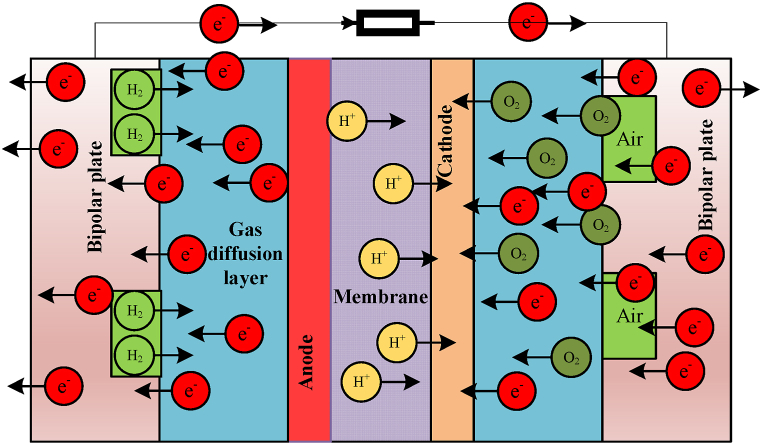
Generalized representation of a Proton Exchange Membrane fuel cell.

In this context, $R_{m}$ is the membrane resistance, and the symbol λ denotes the control parameter, whereas Rc indicates the connection resistance. Fig. (1) depicts a generalized representation of a Proton Exchange Membrane (PEM) fuel cell.

As can be observed from Fig. (1), a visual representation of the primary constituents and their configuration inside a conventional Proton Exchange Membrane (PEM) fuel cell system is given. Located at the focal point of the figure is the Proton Exchange Membrane (PEM) itself, serving as an electrolyte membrane. This mechanism facilitates the preferential permeation of protons while impeding the movement of electrons, so it guarantees the essential ion exchange phenomenon.

The anode and cathode areas are located on opposite sides of the Proton Exchange Membrane (PEM) fuel cell. The anode is located on the left side of the figure, which serves as the point of entry for the fuel, often hydrogen gas. Subsequently, the fuel undergoes a catalytic process inside the anode catalyst layer, resulting in the generation of protons ($H^{+}$) and the liberation of electrons.

The movement of these electrons gives rise to an electric current that may be used for several purposes. Transitioning to the right portion of the figure, the cathode can be seen. In this context, the oxidant is often identified as oxygen derived from the surrounding atmosphere. The oxidizing agent has a reaction with the protons that have traversed the proton exchange membrane (PEM), in conjunction with the electrons from the external circuit.

This leads to the production of water as a byproduct. The aforementioned process takes place inside the cathode catalyst layer. Evidently, the electrodes flanking the PEM are outfitted with a thin coating of catalyst material, such as platinum, in order to enhance and accelerate the chemical processes that take place. The catalyst layers serve to augment the fuel cell's efficiency via the acceleration of reaction rates. Furthermore, apart from the fundamental constituents, the diagram incorporates supplementary significant components, namely the bipolar plates. The purpose of these plates is to function as structural supports and facilitate the passage of fuel and oxidant, so it guarantees a consistent distribution across the electrodes.

The maintenance of ideal conditions and overall performance heavily relies on the proper flow of reactants. The arrows shown in the illustration represent the directional flow of various components and the paths of chemical processes taking place inside the fuel cell.

## 3 SqueezeNet

### 3.1 Structure of network

**Fig. 2:**
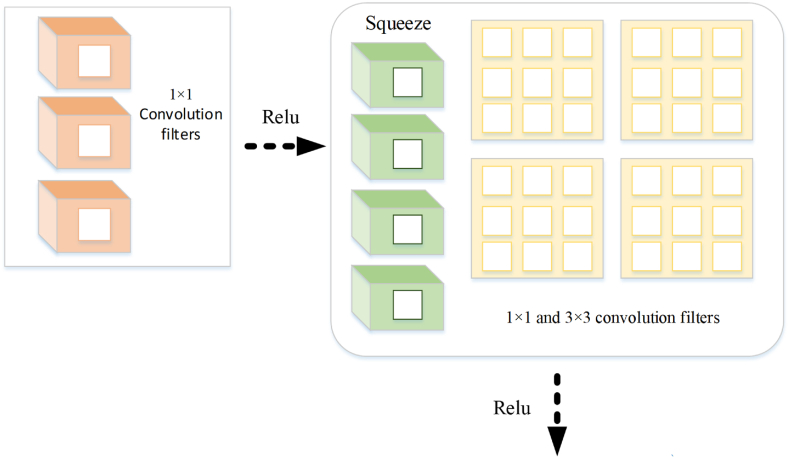
Fire module of the SqueezeNet.

SqueezeNet has been known as a specific kind of Deep Neural Network, which has been made for categorizing image. The primary purpose of it is to preserve good levels of accuracy, while they are effective and smaller compared to other DNNs. There is an approach utilized in SqueezeNet to achieve efficacy and tiny size called Network compression. According to this approach, the SqueezeNet replaces the convolutional layers that are large and expensive with efficient and dense ones. It has been achieved by innovative application through integrating 1×1 convolutions and pooling layers that can extensively decrease the quantity of variables in the network. Moreover, it should be noted that SqueezeNet comprises Fire Modules. The Modules, which are composed of expansion layer and squeeze layer are the building blocks of the network. The Fire Module of the SqueezeNet has been illustrated in Fig. (2).

A 1×1 size Convolutions have been used in the layer of SqueezeNet in order to decrease the quantity of input channels, whereas 3×3 and 1×1 sizes convolutions have been integrated in the layer of expansion for the purpose of raising the quantity of output channels. Eventually, the network can effectively gather global and local input images' properties. Moreover, it should be noted that the Squeeze utilizes a technique called deep supervision, which includes expansion of the network accompanying supplementary classification layers. Having several classification levels offered leads to an increase in the accuracy of the model compared to being dependent on a particular output categorization.

**Fig. 3:**
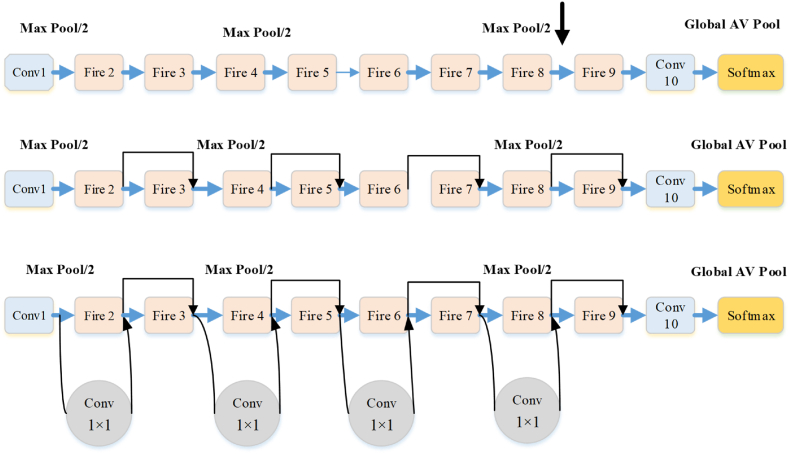
Diagram for Squeeze Net's configuration.

SqueezeNet can be utilized here to model the PEMFC. In order to predict the health model according to its data features, the network might be utilized. Based on the features that has been extracted from the data, the network might receive and predict the model. Deep supervision is able to enhance the accuracy of model that suggests myriad classification layers. The scheme for configuration of SqueezeNet has been illustrated in Fig. (3). The approach of SqueezeNet consists of three discrete phases, comprising eight Fire modules, the convolutional layer, and the ultimate convolutional layer.

### 3.2 Function of fitness

By the use of fitness function, the most meticulous solution of an issue can be found by the function of the Mean Squared Error (MSE). The efficiency of a specific series of variables in deciding the PEMFC identification problem has been evaluated Employing Mean Squared Error. The value of MSE has been ascertained by the approach of MSE as a measurement that how efficient function of the elements is. The value of MSE can assess the average squared distinction between the actual and expected value of an element. The value of MSE can measure the parameters' efficiency.

(17) $$Fit=\frac{1}{N}\sum_{j=1}^{N}{\left(D_{j}-Y_{j}\;\right)}^{2}$$

here, the entire quantity of samples has been defined by N, and the output and the wanted value of the SqueezeNet has been, in turn, denoted by $Y_{j}$ and $D_{j}$. By detecting the series of hyper-parameters that signify the lowest value of MSE like SqueezeNet weights, the optimum solution has been achieved. This specific series of hyper-parameters has been supposed to be the most efficient and meticulous solution of the issue. The central notion has concentrated on the optimum choice of hyper-parameters, which are essential for making the forecasting model. The parameters, which are determined before beginning the training section called Hyper-parameters. In addition, they are able to affect performance of the model extensively. Robustness and accuracy of the model can be increased by choosing the optimum hyper-parameters.

Various optimality techniques, like stochastic gradient descent, can be used for training the network for the purpose of improving the accuracy of the model and decreasing categorization error. Furthermore, metaheuristics has been known as a novel popular strategy for this aim.

A major advantage of using meta-heuristic for the optimum ascertaining weights of SqueezeNet is that it can effectively and efficiently discover a large area of likely values of weight. Because there are many weights in the model of SqueezeNet that should be optimized, it is a factor that is of paramount significance; the reason is to gain optimum performance. An improved meta-heuristic is employed in this study, which is called Modified Transient Search Optimization Algorithm. This method will be completely described, subsequently.

## 4 The modified transient search optimization algorithm

### 4.1 Transient search optimization algorithm

**Fig. 4:**
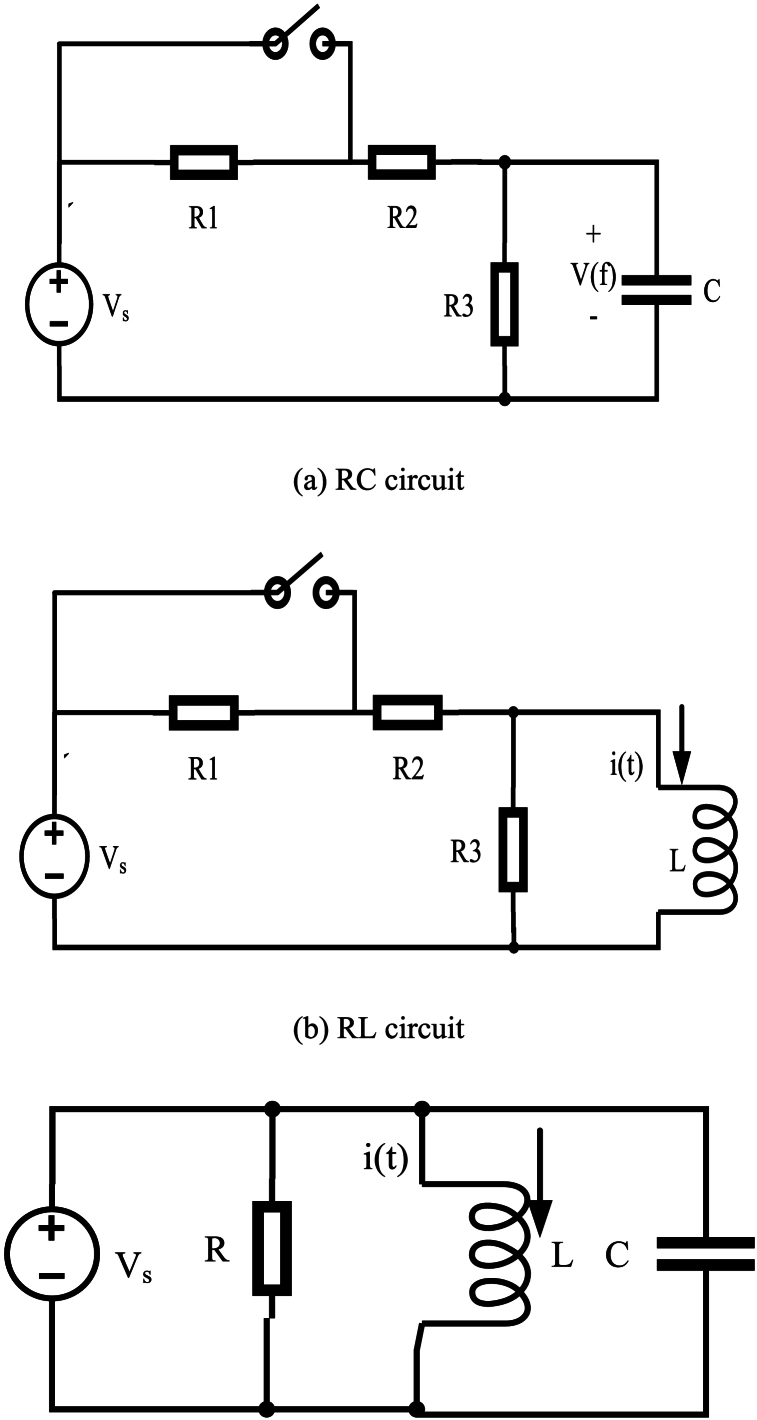
Second order circuit (RLC).

The electrical circuits have Resistive (R) and other constituents of electricity storage, including Inductor and Capacitor (LC), Inductor (L), and Capacitor (C). The circuit comprises a stable response, which has been known as the last response and a response transient. It has been fully illustrated in Eq. (19). Fig. (4) shows that circuits with only one storing constituent (RL or RC) are referred to as first-order circuits, whereas Fig. (4) illustrates that circuits with two storage constituents (RLC) are called second-order circuits.

**Fig. 5:**
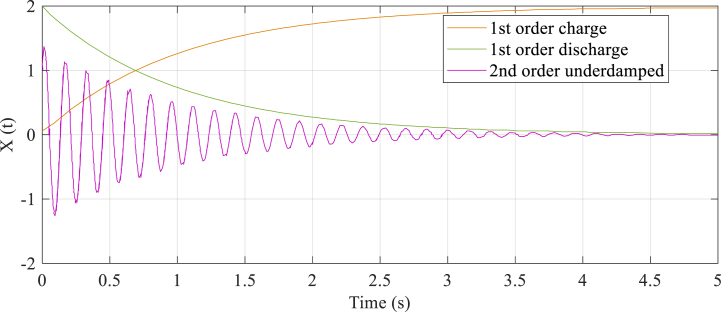
Transient response of second-order and first-order circuits.(18)Completeresponse=Transientresponse+Finalresponse(19)$\frac{d}{dt}x\left(t\right)+\frac{x\left(t\right)}{T}=G$(20)$x\left(t\right)=x\left(f\right)+\left(x\left(I\right)-x\left(f\right)\right)e^{\frac{-1}{T}}$

When circuits are switched, the process does not immediately move to the next stable-state. This is because capacitors or inductors need time to charge or discharge before reaching their stable-state value. It is illustrated in Eq. (20) that various equations can calculate first-order circuit transient response. In order to discover the solution x(t), various formulations can be solved, which has been illustrated in Eq. (21). In Fig. (5), the exponential response for discharging and charging operations is displayed as the first-order circuit transient response.

here, time is denoted by t, then it is possible to use x(t) as either the RL circuit's inductor current i(t) or the RC circuit's capacitor voltage v(t). This can provide valuable insight into the behavior of the circuit and aid in analysis and design. where, time constant of the circuit is denoted by T, τ=RL circuit's L/R and τ=RC circuit's RC. K is a constant that depends on the initial value x(I); moreover, the value of the final response is denoted by x(f). The difference formulation, which has been shown by Eq. (22), can calculate the second-order circuit's transient response. Eq. (23) illustrates second-order difference equation's solution. The RLC circuit response is classified as under-damped.

(21) $$\frac{d^{2}}{dt^{2}}x\left(t\right)+2\alpha \frac{d}{dt}x\left(t\right)+w_{0}^{2}x\left(t\right)=f\left(t\right)$$

(22) $$x\left(t\right)=e^{-\alpha t}\left(E_{1}\;\cos\left(2\pi f_{d}t\right)+E_{2}\;\sin\left(2\pi f_{d}t\right)\right)+x\left(f\right)$$

The parameters $\alpha ,w_{0},f_{d},E_{1}\text{,}$ and $E_{2}$ are, in turn, denoted by damping coefficient, frequency of resonant, the frequency of the damped resonant, and the last two ones are constant. When the value of α is less than $w_{0}$, the RLC circuit's transient response shows damped oscillations, which is known as under-damped response. Fig. (5) illustrates this phenomenon.

### 4.2 Inspiration of the TSO algorithm

In this phase, there are some stages in TSO algorithm which are: A) initializing the agents of search between upper and lower bounds in the search space; B) searching the finest solution which is called Exploration; and reaching the finest solution which is called Exploitation. The first stage is produced randomly just like mentioned in Eq. (24). In the second stage, the second-order RLC circuits' oscillations inspire TSO's exploration manner which is around zero. Moreover, it has been fully depicted in Fig. 3. But the first-order discharge's exponential decaying inspires TSO's exploitation manner that has been thoroughly illustrated in Fig. (5). Exploitation $\left(r_{1}\;<\;0.5\right)$ and the exploration $\left(r_{1}\geq 0.5\right)$ of the TSO algorithm have been balanced by $r_{1}$ which denotes the random number. Eq. (25) displays calculated design of the exploration and exploitation of the TSO. It should be noted that Eq. (23) and Eq. (21) has inspired Eq. (25). The TSO's finest solution $Z_{l}^{*}$ depicts electrical circuit's final value (x(f)), and $E1=E2=|Z_{l}-C_{1}\times Z_{l}^{*}|$.

(23) Z=lb+rand×(ub−lb)

(24) $$Z_{l+1}=\left\{ \begin{array}{c} Z_{l}^{*}-C_{1}\times Z_{l}^{*})e^{-T},r_{1}<0.5\\ Z_{l}^{*}+e^{-T}[\cos\left(2\pi T\right)+\sin\left(2\pi T\right)\left|Z_{l}-C_{1}\times Z_{l}^{*}\right|,r_{1}\geq 0.5 \end{array}\right.$$

(25) $$T=2\times z\times r_{2}-z$$

(26) $$C_{1}=k\times z\times r_{3}+1$$

(27) z=2−2(lLmax)

The search space's lower bound and upper bound are, in turn denoted by lb and ub, while the random number, which has been distributed uniformly, is denoted by rand, and z has been considered a parameter which is in the range of 0–2 illustrated in Eq. (28). $r_{1},r_{2}$, and $r_{3}$ are random numbers which have been distributed uniformly between [0,1], random coefficients are denoted by T and $C_{1}$, the location of agents for search is denoted by T and $Z_{l}$, the finest position has been denoted by $Z_{l}^{*}$, the iteration number has been denoted by l, the maximum number of iteration has been denoted by $L_{\max}$, and k has been known as a number which is constant (k=0,1,2,…).

**Fig. 6:**
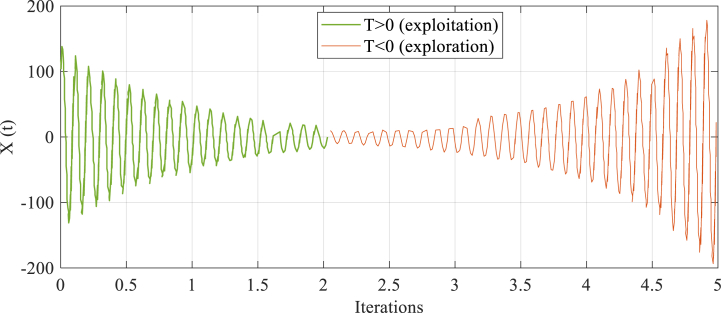
Exploitation and exploration process.

Moreover, coefficient T realizes the balance between exploitation and exploration process that is various from −2 to 2. Fig. (6) illustrates that when T>0, the exploitation phase of the algorithms can be gained. Whereas, the exploration phase is obtained when T<0. Fig. (6) clearly shows that the transient response begins with a high value, then it gradually decreases to its lowest point when T is greater than 0. Next, it oscillates and rises to higher values when T becomes less than 0.

It is clear that the algorithm being proposed is not very complicated, as it only uses one formulation to update position and balances exploitation and exploration procedures. First, the finest position and the population $Z_{l}^{*}$ and $Z_{l}$ must be initialized. Then, the benchmark of the population must be assessed. If $l<\;L_{\max}$, the values of $C_{1}$ and T must be updated by the use of Eq. (27) and Eq. (26). Next, the location of population must be updated using Eq. (25).

In addition, the benchmark function of the new members of population must be calculated, and if the prior finest benchmark function is more than the current one, the finest value must be updated. Furthermore, the mathematical complication of the TSO has been computed by the use of big-oh notation. At first, TSO algorithm process initializes agents of search and assesses them by the use of benchmark function. After that, the search agents must be upgraded in accordance with the function assessment.

### 4.3 Modified transient search optimization algorithm (MTSO)

The Transient Search Optimization Algorithm (TSOA) is a metaheuristic algorithm used to solve optimization issues, drawing inspiration from dynamical systems. It is a simulation-based approach that explores the search space and iteratively evaluates transient states until an optimum solution is identified. Modified TSOA can improve convergence speed, handle restrictions, and achieve a harmonious balance between exploration and exploitation. Integrating issue-specific information can also enhance performance, depending on the problem being addressed. TSOA may experience degradation when faced with optimization issues with large dimensions, but various approaches can be used to address these limitations. Overall, TSOA modification allows for customization to accommodate specific optimization issues, enhance efficiency, and overcome constraints for superior outcomes. In this study, two modifications, including Lévy flight mechanism and self-adaptive population changes, are used for this purpose.

The Lévy flight mutation refers to a kind of random walk characterized by size of step that adheres to a probability distribution with large tails. The exploration capabilities of the TSOA may be enhanced by including the Lévy flying mutation, which enables extended leaps inside the search space. This approach has the potential to mitigate the challenges posed by local optima and expedite the process of convergence.

In the search process, at each iteration, a stochastic sampling method may be used to choose a subset of people from the population. The placements of these people may thereafter be disrupted via the use of Lévy flight stages. The introduction of this mutation incorporates stochasticity and extended displacements, hence enhancing the algorithm's capacity to efficiently explore various locations within the search space.

The updated finest solution for the proposed modified TSO combining with the Lévy flight can be achieved as follows:

(28) T=2×z×fx−z

(29) $$C_{1}=k\times z\times fx+1$$

where, fx is the Levy distribution and is achieved by the following equation:

(30) $$fx=\frac{1}{\sqrt{2\pi \sigma^{2}}}\exp\left(-\frac{\gamma }{2\sigma^{2}}\right)x-\mu \frac{\gamma }{x-\mu^{1+\gamma }}$$

where, σ is a scale parameter that represents the standard deviation of the distribution. μ represents the location, which corresponds to the mean of the distribution, γ is the tail index parameter, which governs the form of the distribution, and σ is a scale parameter that represents the standard deviation of the distribution.

The shape of the distribution is determined by the tail index parameter γ. When the parameter γ falls between the range of 0 and 2, the distribution exhibits infinite variance, indicating that the variance of the distribution cannot be determined. When the value of γ exceeds two, the distribution exhibits a finite variance, but if γ is less than or equal to 1, the distribution demonstrates an infinite mean.

The optimization process in TSOA maintains a constant population size, without any self-adaptive changes. Nevertheless, under some circumstances, the act of dynamically modifying the population size has the potential to provide more favorable outcomes. The use of self-adaptive population modifications enables the algorithm to dynamically adjust the population size in response to the prevailing conditions of the search process.

One such strategy is implementing a system that regularly assesses the performance of the population. In the event that the population reaches a state of stagnation or fails to exhibit improvement, the algorithm has the capability to augment the population size as a means to bring a greater degree of variety and facilitate exploration. In contrast, in the event that the population exhibits rapid convergence, the algorithm may choose to decrease the population size in order to prioritize exploitation. The population modifications may be executed repeatedly or activated by certain situations, hence enabling a self-adaptive behavior that enables TSOA to dynamically modify its exploration-exploitation balance. The initial population size prior to the establishment of the algorithm's primary loop is determined as follows:

(31) Size(Z)=10×dim

where, dim describes the problem dimension.

The updated population size can be determined using the following method:

(32) $$Siz{e\left(Z\right)}_{new}=round\left(Size\left(Z\right)+\theta \times Size\left(Z\right)\right)$$

The parameter θ represents a discrete random variable that takes on integer values between −0.5 and 0.5. The possible influence of this factor on the size of the population is projected to result in a change of around 45%, either in the form of an increase or a decrease from the existing population level. Based on the reference provided, it is indicated that the population has the ability to fluctuate by around 45% in either a positive or negative direction. In the event that the population size in the subsequent epoch, denoted as $Size{\left(Z\right)}_{new}$, surpasses the population size in the preceding epoch, denoted as Size(Z), whole of the individuals from the present population are preserved, and a novel population is generated by the application of elitism. The selection of optimal replies is made from the previous iteration. In cases when the population size declines compared to the previous epoch ($Size{\left(Z\right)}_{new}<Size\left(Z\right)$), only the individuals with the highest level of success are retained, while those who did not perform well are destroyed.

### 4.4 Verification of the proposed MTSO metaheuristic algorithm

In order to assess the performance and effectiveness of the newly designed metaheuristic algorithm named MTSO (Transient Search Optimization with Lévy Flight Mutation and Self-Adaptive Population Changes), it is essential to conduct a rigorous validation process [23]. The verification involves comparing MTSO with five other established optimization methods on a selection of standard benchmark functions taken from the “CEC-BC-2017 test suite” [24]. This section outlines the verification procedure and presents the obtained results. The selected functions ensure a proper representation of complexity in landscape problem [25].

Five widely-used optimization methods are chosen as benchmarks to be compared with MTSO. These methods may include Jaya Algorithm, Monarch Butterfly Optimization (MBO), Slime Mould Algorithm (SMA), Elephant Herding Optimization (EHO), and Water Wave Optimization (WWO). The selection is based on their popularity and success in solving various optimization problems. Table 1 indicates the parameter configurations for various metaheuristic algorithms.

**Table 1:** Parameter Configurations for various metaheuristic algorithms.

Algorithm	Parameter and value
Jaya Algorithm (JA)	N = 30, T = 100
Monarch Butterfly Optimization (MBO)	N = 30, T = 100, Pp = 0.5
Slime Mould Algorithm (SMA)	N = 30, T = 100, α = 2
Elephant Herding Optimization (EHO)	N = 30, T = 100, α = 0.1, β = 0.1
Water Wave Optimization (WWO)	N = 30, T = 100, α = 0.5, $h_{\max}$ = 0.1

The proposed MTSO and the comparison methods are implemented using MATLABR2019b, and other methods are set as recommended in the literature or fine-tuned through preliminary experiments. Each method is executed multiple times (e.g., 25 runs) to account for the stochastic nature of metaheuristics. The termination criterion is set as a maximum number of iterations or a predefined tolerance value. The experiments are conducted on a sufficiently powered computing system to ensure fair execution times for all methods.

The assessment of algorithmic efficacy in this research is based on three primary metrics: Mean, standard deviation (StD), and the optimal values attained by the examined functions. The metrics were calculated 20 times by considering their average value and by considering 200 iteration for each algorithm. Table 2 presents a comparative study of the MRPO algorithm, as described in this research work, compared to other examined algorithms. This section shows the findings derived from the evaluation of the algorithms within the framework of the study. Table 2 presents a comparative analysis of the execution results of the different algorithms under investigation and the proposed MTSO method.

**Table 2:** Comparison of optimization algorithms' performance on CEC-BC-2017 test suite based on Best, Mean, and Standard Deviation.

Function	Indicator	MBO	JA	SMA	EHO	WWO	MTSO
F1	Best	12.71	14.74	10.14	112.63	3.55	1.35
	Mean	16.29	1.55	18.74	13.18	1.94	2.58
	StD	12.14	139.42	11.93	14.74	14.51	8.79
F2	Best	0.37	7.19	78.54	6.73	4.83	0.48
	Mean	32.39	50.01	0.56	59.90	0.48	32.63
	StD	0.26	33.26	41.92	41.92	32.39	0.56
F3	Best	0.04	40.42	36.11	17.48	32.03	0.00
	Mean	36.11	17.48	0.03	0.00	32.03	0.01
	StD	0.01	8.64	0.01	12.09	10.22	0.00
F4	Best	0.43	6.82	8.06	4.98	7.07	0.15
	Mean	7.07	4.98	0.00	8.06	7.00	0.12
	StD	0.08	1.71	0.16	1.78	1.79	0.00
F5	Best	0.00	0.20	4.02	2.32	4.92	0.00
	Mean	4.02	2.00	0.01	0.00	4.42	0.00
	StD	0.00	1.27	0.00	1.17	1.86	0.00
F6	Best	0.00	1.52	1.32	0.11	0.15	0.00
	Mean	1.52	1.16	0.94	0.15	0.00	0.00
	StD	0.00	2.21	1.77	1.60	0.00	0.00
F7	Best	0.90	0.03	1.29	0.47	1.46	0.40
	Mean	1.29	1.67	1.70	0.55	0.83	1.84
	StD	0.21	0.19	0.25	0.30	0.19	0.21
F8	Best	9.94	13.55	8.31	11.53	19.91	7.39
	Mean	22.43	21.16	13.72	13.19	18.87	19.91
	StD	3.80	6.81	6.65	0.20	5.01	5.52
F9	Best	11.74	0.00	32.03	0.00	0.00	0.20
	Mean	32.03	23.70	0.00	0.00	2.59	0.00
	StD	0.00	10.56	0.00	1.71	20.14	0.00
F10	Best	4.22	0.16	4.15	79.11	2.03	4.57
	Mean	8.72	195.00	2.53	9.06	11.46	4.15
	StD	4.27	5.21	5.69	6.28	6.12	28.94
F11	Best	0.10	0.06	0.15	0.00	0.01	0.14
	Mean	0.29	0.16	0.15	0.00	0.01	0.16
	StD	0.00	0.13	0.09	0.08	0.00	0.00
F12	Best	0.00	0.00	0.00	0.00	0.00	0.00
	Mean	0.00	0.00	0.00	0.00	0.00	0.00
	StD	0.00	0.00	0.00	0.00	0.00	0.00

The obtained results for each benchmark function are presented and discussed comprehensively. Statistical analyses, such as mean, standard deviation, and significance tests (e.g., t-tests), are performed to compare the performance of MTSO with other methods. Table 2 is utilized to facilitate the interpretation of the results. Here, the performance of MTSO is critically analyzed and compared with other methods. The strengths and weaknesses of MTSO are discussed based on the experimental outcomes. Insights into the algorithm's behavior and its suitability for different types of problems are provided.

## 5 Simulation results

### 5.1 Model configuration

The training dataset was prepared for the proposed technique to evaluate the efficiency of a Proton Exchange Membrane Fuel Cell (PEMFC) stack. The stack configuration, operating conditions, control parameters, and performance metrics were chosen to ensure accurate evaluation and validation. These settings included the number and arrangement of fuel cells, temperature, pressure, humidity, fuel flow rate, air supply, cooling, and voltage regulation. The efficiency of the technique was measured using relevant metrics, such as power output, current density, and voltage efficiency. By utilizing these settings, the efficiency of the proposed technique was accurately evaluated and validated, ensuring that it effectively optimizes the performance of the PEMFC stack based on the given operating conditions and control parameters. In Table 3, various parameters and corresponding values for a fuel cell system are presented. The table provides important information about the system's characteristics and operational conditions.

**Table 3:** Parameters and values for a Fuel Cell system [26].

Parameter	Value	Unit	Parameter	Value	Unit
$RH_{c}$	1	–	n	26	–
$RH_{a}$	1	–	T(K)	350.15–360.15	K
Membrane thickness	132	μm	Rated Power	260	W
Cell operating current	30	$cm^{2}$	$P_{a}$	1–3	bar
Maximum current density	875	$A\;cm^{-2}$	$P_{c}$	1–5	bar

Verification considers four operational conditions: 3/5 bar, 1/1 bar, 2.5/3 bar, and 1.5/1.5 bar at 353.15 K. In this study, a dataset comprising 250 pairs of input-output data was utilized. These data points were extracted from the previously mentioned four functional situations, ensuring a diverse representation of operating conditions. To ensure robust training and evaluation, the dataset was divided into training and testing subsets.

The training dataset consisted of 70% of the data, specifically 175 instances. This subset was used to train the planned network, allowing it to learn the underlying patterns and relationships between inputs and outputs. The remaining 30% of the data, equivalent to 75 instances, was reserved for evaluating the network's efficiency and performance.

To comprehensively assess the network's performance, all four main operating conditions, as included in the testing dataset, were used for evaluation. This approach ensured that the network's predictions could accurately account for the variations arising from different operational scenarios.

Moreover, an additional dataset obtained from Ref. [27] was incorporated into the study. This dataset encompassed 60 pairs of data points, collected under diverse operating settings. The purpose of utilizing this dataset was twofold: firstly, to showcase the polarization profiles, which depict the relationship between voltage and current density, and secondly, to evaluate the accuracy of the proposed MTSO-based SqueezeNet model in estimating these profiles.

By encompassing a variety of datasets and operating conditions, this study aimed to enhance the reliability and generalizability of the proposed MTSO-based SqueezeNet model and its estimations of polarization profiles.

In this study, before using the data for training, it was preprocessed by normalizing between 0 and 1. Normalizing the data of a PEMFC facilitates accurate comparisons, improves model performance, ensures convergence, aids interpretability, and mitigates the impact of outliers. It enables a more reliable and consistent analysis of the fuel cell system. When normalizing data between 0 and 1 in PEMFC stack modeling, there are several options to consider. This research used Z-Score Normalization for this purpose.

Z-Score Normalization, sometimes referred to as normalization, is a data transformation process that involves removing the mean value from each data point and then dividing the result by the standard deviation. The mathematical expression for doing Z-score normalization is as follows:

(33) $$x_{z}=\left(x-mean\right)/\sigma$$

where, x defines the basic data, and mean and σ represent the mean value and the standard deviation value for the data. Z-score normalization centers the data around 0 and scales it based on the standard deviation.

To achieve normalization between 0 and 1 using Z-Score normalization, an additional normalization stage is required. This can be done using the following formula:

(34) $$x_{Norm}=\left(x-\underline{x}\right)/\left(\bar{x}-\underline{x}\right)$$

where, $\underline{x}$ and $\bar{x}$ represent the minimum and maximum value of the data, respectively.

### 5.2 Results and discussions

**Fig. 7:**
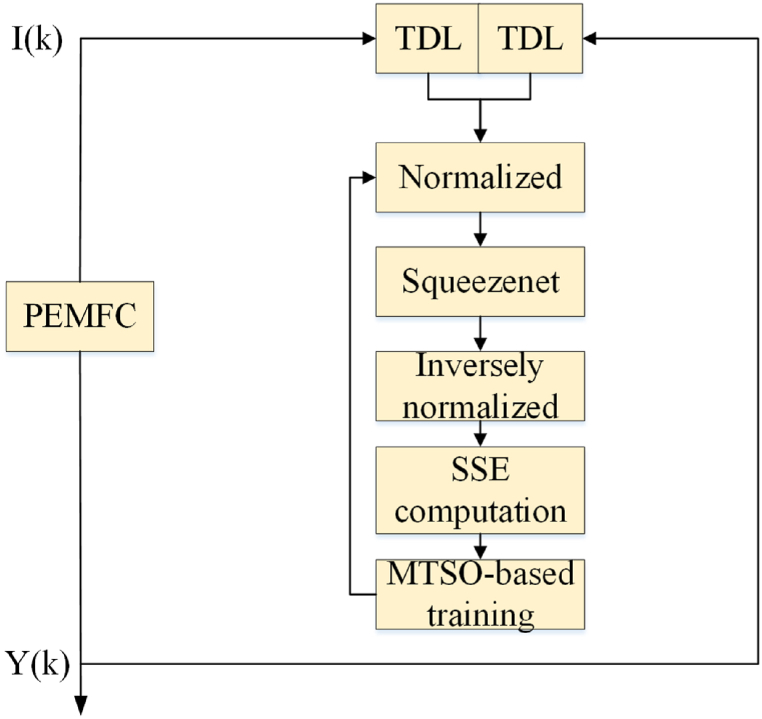
Model determination of a Proton Exchange Membrane (PEM) Fuel Cell using a MTSO-based SqueezeNet.

This section shows the outcomes and analyses of our simulation using the proposed Multi-Terminal Switching Optimization (MTSO)-based SqueezeNet model on the previously described dataset. The objective of the simulation is to predict the future output voltage values by using the preceding voltage and current values. Fig. (7) showcases the model determination of a Proton Exchange Membrane (PEM) Fuel Cell using a MTSO-based SqueezeNet.

As shown in Fig. (7), the Time Delay Line (TDL) block plays a crucial role in the optimal configuration design of the proposed MTSO-based SqueezeNet. The objective function of this system is to minimize the Sum of Squared Errors (SSE) between the real output voltage of the PEM Fuel Cell and the predicted output voltage of the proposed network.

The TDL block introduces a time delay element which takes into account the temporal dynamics and delays in the PEM Fuel Cell system. This is important because the output voltage of the fuel cell is influenced by various factors, such as changes in operating conditions, load demands, and system response time. By incorporating a time delay, the proposed MTSO-based SqueezeNet can effectively capture and account for these dynamics, leading to an improved accuracy in voltage prediction.

The SSE is used as the objective function for configuring the optimal parameters of the network. The SSE represents the cumulative error between the actual output voltage and the predicted output voltage for a given set of input data. Minimizing the SSE ensures that the predicted values closely match the ground truth values, indicating a high level of accuracy and precision in the model's predictions.

By optimizing the configuration of the MTSO-based SqueezeNet using the TDL block and minimizing the SSE, an optimal network structure can be achieved that can accurately forecast the output voltage of the PEM Fuel Cell. This is crucial for the effective monitoring, control, and optimization of fuel cell systems in real-world applications. The SSE can be mathematically defined as follows:

(35) $$SSE=\min\left(\sum_{i=1}^{M}{\left(Z-\hat{Z}\right)}^{2}\right)$$

The sampling amount of empirical information is defined by the parameter M. The objective of the research was to minimize the Sum of Squared Errors (SSE) using the suggested Multi-Terminal Switching Optimization (MTSO) algorithm. This algorithm was employed to determine the centers of unseen neurons in the SqueezeNet model.

To provide a proper validation, its results were compared with two other state of the art methods from the literature, including the Gated Recurrent Unit and Improved Manta Ray Foraging Optimization (GRU/IMRFO) [18] and Grey Neural Network Model integrated with Particle Swarm Optimization (GNNM/PSO) [19].

The GRU/IMRFO algorithm is a deep learning model that combines the power of the two-dimensional Convolutional Neural Network (CNN2D), the Gated Recurrent Unit (GRU), and the Improved Manta Ray Foraging Optimization (IMRFO) algorithm. By leveraging the CNN2D, the algorithm is able to extract spatial features from the PEMFC data, while the GRU captures the temporal dependencies of the PEMFC degradation. Additionally, the IMRFO algorithm optimizes the parameters and structure of the neural network. This unique combination allows the GRU/IMRFO algorithm to effectively handle the nonlinear and complex characteristics of PEMFC performance degradation, resulting in an accurate and reliable predictions.

**Fig. 8:**
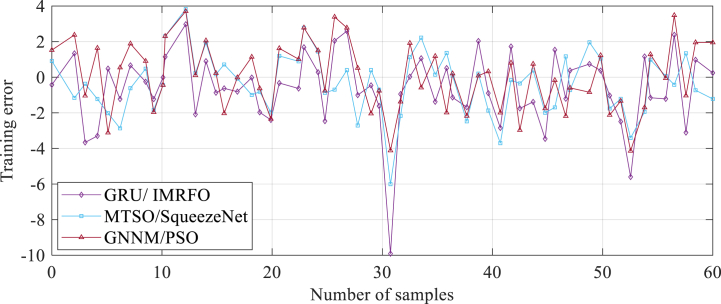
Learning error profile of the data samples used in determining the PEM Fuel Cell characteristics.

On the other hand, the GNNM/PSO algorithm is a grey system model that integrates the Grey Neural Network (GNN) and the Particle Swarm Optimization (PSO) algorithm. The GNN is specifically designed to handle uncertainties and incomplete information present in the PEMFC data, while the PSO algorithm enhances the learning ability and convergence speed of the GNN. As a result, the GNNM/PSO algorithm improves the forecast accuracy and efficiency of the grey system model, providing stable and robust predictions. To evaluate the performance of the proposed model, it was compared with the two aforementioned algorithms. Fig. (8) illustrates the learning error profile of the data samples used in determining the PEM Fuel Cell characteristics.

**Fig. 9:**
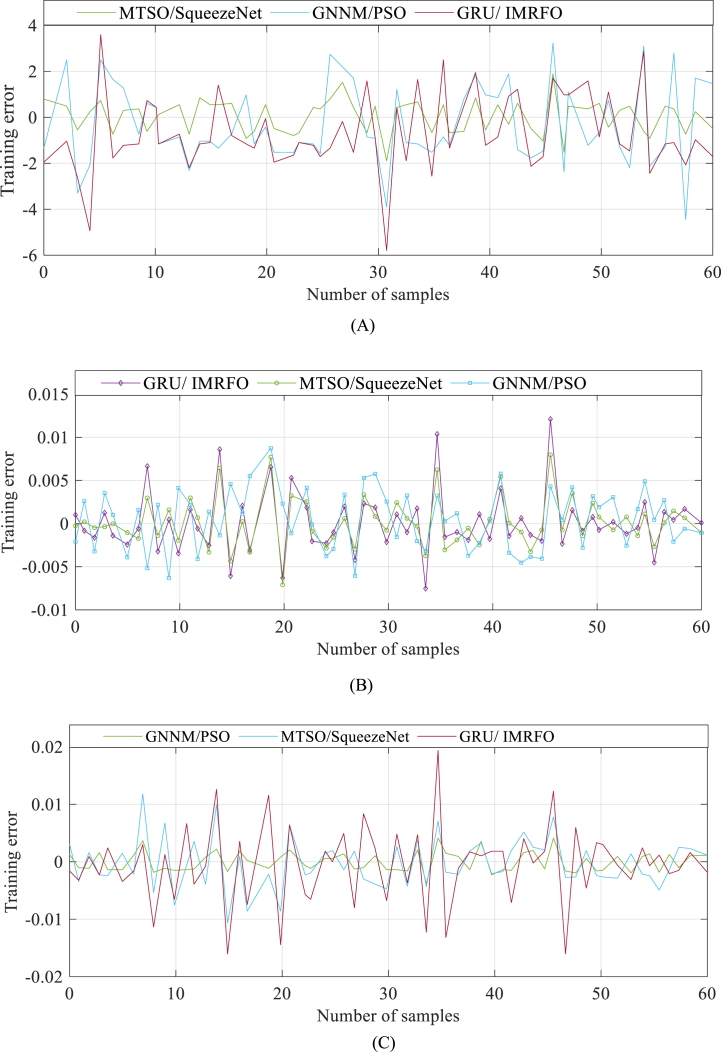

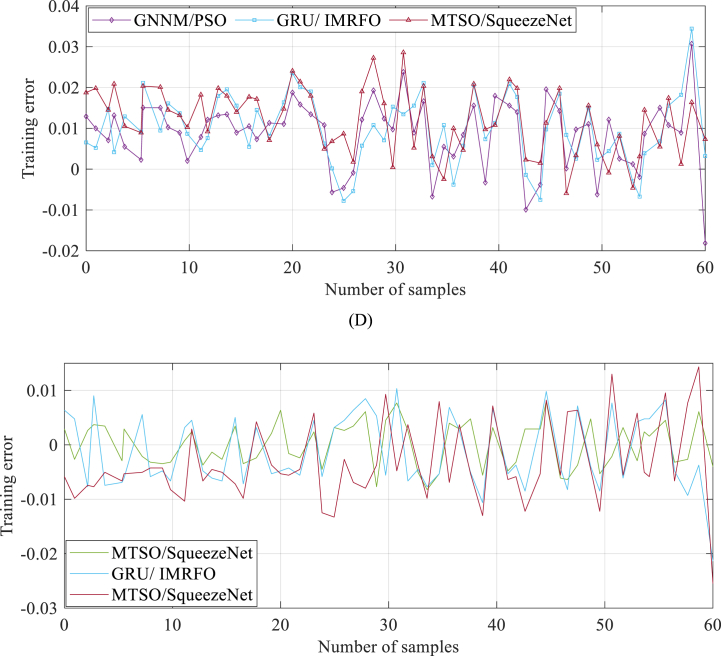
Error profiles during the verification process: (A) 3/5 bar and 353.15 K, (B) 1.1 bar and 343.15 K, (C) 2.5 bar and 343.15 K, and (D) 1.5/1.5 bar and 343.15 K.

As previously mentioned, the trained MTSO-based SqueezeNet model is evaluated to measure its performance. The error profiles during the verification process are depicted in Fig. (9), showcasing the accuracy and performance of the model across different datasets.

As seen in Fig. (9), it is evident that the discrepancy between the experimental values and the projected values is minimal.

The results clearly demonstrate that the proposed model outperforms both algorithms across various test functions and data sets during accurately determining the model parameters. Specifically, during the verification process of 3/5 bar and 353.15 K, 1.1 bar and 343.15 K, 2.5 bar and 343.15 K, and 1.5/1.5 bar and 343.15 K, the proposed model consistently exhibits superior learning error profiles. This highlights the effectiveness and reliability of the GRU/IMRFO algorithm in accurately predicting performance degradation of PEMFC.

**Fig. 10:**
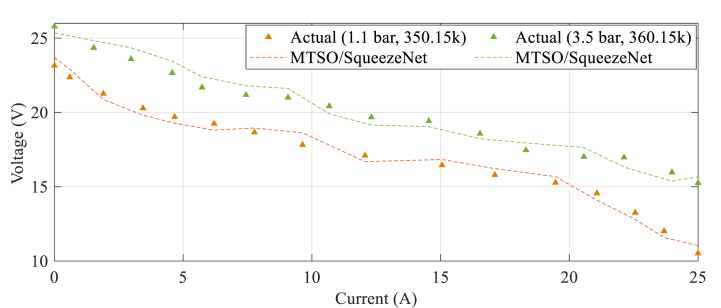
Polarization profiles of the four functional conditions that were analyzed using the proposed MTSO-based SqueezeNet model.

To enhance clarity, the polarization profiles were examined using the MTSO-based SqueezeNet model developed in this study. This analysis aimed to validate the forecasting accuracy of the network, as seen in Fig. (10). The polarization profile represents a graphical representation that illustrates the relationship between the voltage output of a fuel cell and the current density. It serves as a means to evaluate the fuel cell's effectiveness and efficiency across various operational scenarios. The accomplishments are also compared with those data in situation 1.1 bar with 350.15 K and 3.5 bar with 360.16 K in order to demonstrate the improved performance of the recommended approach.

The MTSO-based SqueezeNet model, as demonstrated in Figure (10), exhibits a remarkable ability to closely replicate the actual polarization profiles of the fuel cell across various functional conditions. This model showcases a low Mean Square Error (MSE) and a high coefficient of determination ($R^{2}$) for all conditions. Furthermore, it displays excellent generalization capabilities by accurately predicting polarization profiles for both hydrogen-oxygen and hydrogen-air reactants. In comparison with the conventional SqueezeNet model and other cutting-edge models like GRU/IMRFO and GNNM/PSO, this model surpasses them in terms of prediction accuracy and efficiency. The experiment was conducted on a Proton Exchange Membrane Fuel Cell (PEMFC) system, which is a low-temperature fuel cell utilizing a polymer electrolyte membrane as the electrolyte.

As can be observed from Fig. (10), the proposed SqueezeNet model based on the MTSO learning approach demonstrates efficacy in the context of system identification. It adeptly captures and models the distinctive attributes of a system across various operating situations with a high degree of accuracy. The use of this technique presents enhanced precision, resilience, and computational efficacy, hence establishing it as a prospective strategy for precisely defining and comprehending the dynamics of a system. Additional study and testing are required to investigate the practicality of its implementation in real-world contexts and enhance its potential for evaluating intricate systems. The confirmation of the model's capacity to provide useful insights into system behavior and performance is shown by the current-voltage profile.

## 6 Conclusions

The modeling of Proton Exchange Membrane Fuel Cells (PEMFCs) plays a vital role in the optimization of performance, reduction of costs, enhancement of durability, and integration of the system. Through a thorough comprehension of intricate electrochemical processes, precise models have the potential to enhance the optimization of design and operating conditions, hence resulting in improvements in power production, efficiency, and durability. The use of modeling techniques also facilitates the identification of alternate materials or configurations, hence enhancing the economic feasibility of PEMFCs across diverse applications. Additionally, it aids in the prediction of cell's lifetimes and enhances the implementation of system integration and control mechanisms. The use of design optimization techniques, such as rapid prototyping and iterative testing, has been shown to effectively decrease both development time and costs. The present research work aimed to address the challenge of model identification of PEMFCs by proposing an improved approach. The researchers utilized the modified Squeezenet model, a deep learning architecture known for its computational efficiency and compact size, as the basis for their modeling framework. Additionally, they incorporated the Modified Transient Search Optimization Algorithm to optimize the Squeezenet configuration specifically for modeling PEMFCs. The modified Squeezenet model was chosen for its ability to capture the complex dynamics and behaviors of PEMFCs while minimizing computational resources. By customizing the architecture and training it with relevant data, the researchers were able to enhance its accuracy and applicability to the specific requirements of PEMFC modeling. To validate the effectiveness of the proposed system, extensive simulations were conducted. The simulation results were compared with empirical data obtained from experimental setups, as well as some other methods, including Gated Recurrent Unit and Improved Manta Ray Foraging Optimization (GRU/IMRFO) and Grey Neural Network Model integrated with Particle Swarm Optimization (GNNM/PSO). The comparison demonstrated that the modified Squeezenet model combined with the Modified Transient Search Optimization Algorithm outperformed the other methods in accurately representing the behavior of PEMFCs.

## CRediT authorship contribution statement

**Rulin Duan:** Formal analysis, Data curation, Conceptualization. **Defeng Lin:** Formal analysis, Data curation, Conceptualization. **Gholamreza Fathi:** Formal analysis, Data curation, Conceptualization.

## Declaration of competing interest

The authors declare that they have no known competing financial interests or personal relationships that could have appeared to influence the work reported in this paper.
